# Case Report: A Novel Compound Heterozygous Mutation in *IL-10RA* in a Chinese Child With Very Early-Onset Inflammatory Bowel Disease

**DOI:** 10.3389/fped.2021.678390

**Published:** 2021-05-25

**Authors:** Fang Dong, Fangfei Xiao, Ting Ge, Xiaolu Li, Wuhen Xu, Shengnan Wu, Ting Zhang, Yizhong Wang

**Affiliations:** ^1^Department of Gastroenterology, Hepatology and Nutrition, Shanghai Children's Hospital, Shanghai Jiao Tong University, Shanghai, China; ^2^Molecular Diagnostic Laboratory, Shanghai Children's Hospital, Shanghai Jiao Tong University, Shanghai, China; ^3^Institue of Pediatric Infection, Immunity and Critical Care Medicine, Shanghai Children's Hospital, Shanghai Jiao Tong University School of Medicine, Shanghai, China

**Keywords:** VEO-IBD, IL10RA, compound heterozygote mutation, whole-exome sequencing, STAT3 phosphorylation analysis

## Abstract

Very early-onset inflammatory bowel disease (VEO-IBD) is defined as IBD diagnosed in children younger than 6 years of age. VEO-IBD is often associated with a monogenic etiology or primary immune deficiency. Here, we report the case of a 7-month-old Chinese girl diagnosed with VEO-IBD who had a variant in the interleukin-10 receptor A (*IL-10-RA*) gene. The patient presented with recurrent fevers, abdominal pain, diarrhea, perianal abscesses, and oral ulcers. Whole-exome sequencing (WES) identified a novel compound heterozygote mutation, c.395T>G (p.Leu132Arg)/ex.1del (p.?), in the *IL-10RA* gene of the patient. The missense mutation c.395T>G (p.Leu132Arg) was inherited from her mother, and ex.1del (p.?) was inherited from her father. Neither mutation has been reported previously. The IL-10RA function of the patient was defective, as demonstrated by a failure of signal transducer and activator of transcription 3 (STAT3) activation in peripheral blood mononuclear cells (PBMCs) stimulated with recombinant IL-10. The patient underwent matched unrelated peripheral blood hematopoietic stem cell transplantation (HSCT), and the clinical manifestations were dramatically improved. In summary, we identified a novel compound heterozygote mutation, c.395T>G (p.Leu132Arg)/ex.1del (p.?), in *IL-10RA* that caused VEO-IBD in a Chinese child, which further expands the mutational spectrum of *IL-10RA*.

## Introduction

Inflammatory bowel disease (IBD) is a group of chronic inflammatory disorders of the gastrointestinal tract that can occur at any age; these disorders include ulcerative colitis (UC), Crohn's disease (CD), and indeterminate colitis (1). Very early-onset IBD (VEO-IBD) is defined as IBD diagnosed in children younger than 6 years of age, which constitutes ~6 to 15% of the pediatric IBD population (2). Children with VEO-IBD experience more severe gastrointestinal symptoms than those with late-onset IBD developing in adolescents and adults, such as extensive colonic involvement, severe colitis with hematochezia, and perianal abscess and fistulae (3). The etiology of IBD is multifactorial and complex, and VEO-IBD is often associated with monogenic defects or primary immune deficiency (3, 4). In recent decades, a number of monogenic defects have been identified in patients with VEO-IBD (3, 5–7).

Interleukin-10 (IL-10) is an anti-inflammatory cytokine secreted by a range of immune cells and plays an important role in mucosal homeostasis (8). IL-10 binds to a heterodimeric cell surface complex composed of IL-10 receptor A (IL-10RA) and IL-10RB chains, which activates Janus tyrosine kinase-1 (JAK1) and tyrosine kinase-2 (TYK2) (8). The activation of JAK1/TYK2 leads to the phosphorylation of signal transducer and activator of transcription 3 (STAT3) and subsequently induces downstream anti-inflammatory gene expression and exerts anti-inflammatory functions (8). Recent studies (3) demonstrated that monogenic defects in either *IL-10* or *IL-10R* led to the development of aggressive IBD in the youngest children. Patients with IL-10RA deficiency account for ~10% of VEO-IBD, and novel mutations are constantly identified (3, 9). Here, we report a Chinese child with VEO-IBD caused by a novel compound heterozygous mutation in *IL-10RA*. The clinical features and genetic variants of the patient were described in the study, and the loss of function of IL-10RA was determined by functional testing through STAT3 phosphorylation analysis in peripheral blood mononuclear cells (PBMCs) from the patient.

## Case Presentation

A 7-month-old girl presented to the Department of Gastroenterology because of recurrent episodes of fever, abdominal pain, diarrhea, and failure to thrive for 4 months. The girl was born at 40 weeks with a birth weight of 3,000 g. The family history was unremarkable. At the age of 3 months, she was admitted to the pediatric intensive care unit (PICU) due to 8 days of unexplained fever, vomiting, severe diarrhea, spasmodic cough, and aggravated shortness of breath for half a day. The patient was given intravenous administration of broad-spectrum antibiotics for clinical sepsis and supportive treatment for other symptoms in the PICU. After discharge from the PICU, the patient still suffered from multiple episodes of fever, diarrhea, and failure to gain weight. On admission, she had a weight of 4.3 kg (<1st percentile, Z score, −5.10) and a height of 56 cm (<1st percentile, Z score, −5.20). Diffuse small miliary red papules around the skin of the mouth and a white ulcer (3^*^2 mm) on the right-side buccal mucosa of the mouth were observed. Furthermore, she suffered from perianal ulcers and vulvar abscesses. Laboratory tests showed low levels of total protein (44.89 g/L, reference range: 60–80 g/L) and albumin (25.13 g/L, reference range: 38–54 g/L), elevated white blood cells (WBCs, 17.89^*^10^∧^9/L, reference range: 8–12^*^10^∧^9/L), an elevated erythrocyte sedimentation rate (ESR, 120 mm/h, reference range: 0–20 mm/h), and elevated procalcitonin (PCT, 8.98 ng/ml, reference range: <0.1 ng/ml), and C-reactive protein (CRP, 89 mg/L, reference range: <5 mg/L) levels. The liver biochemical profile was normal. Lymphocyte subset analysis was normal. Slightly elevated levels of immunoglobulin G (IgG), IgA, IgM, and IgE were detected. Endoscopy and colonoscopy were abandoned due to the low weight of the patient. An abdominal computed tomography (CT) scan revealed bowel wall thickening and pneumatosis. The patient was treated with montmorillonite powder, total parental nutrition (TPN), methylprednisolone, thalidomide, and intravenous administration of broad-spectrum antibiotics. She was discharged with some symptomatic improvement. However, the patient still suffered from recurrent episodes of fever, abdominal pain, and diarrhea. Therefore, she was suspected to have a primary immunodeficiency disease and was recommended for genetic testing.

### Identification of a Novel Compound Heterozygous Mutation in *IL-10RA*

**Figure 1:**
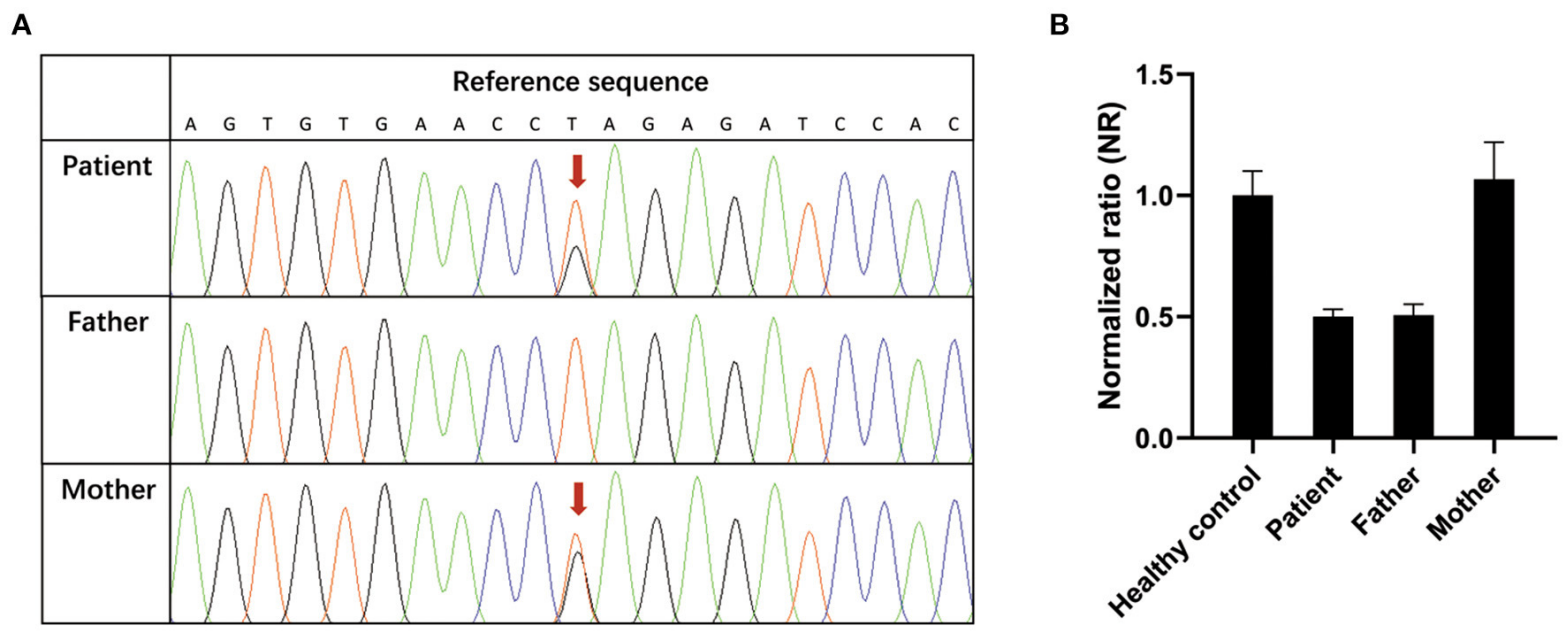
Confirmation of novel IL-10RA variants. **(A)** c.395T>G variant (red arrow) confirmed by Sanger sequencing. **(B)** Validation of the ex.1del (p.?) variant by quantitative PCR. Genomic DNA was extracted from peripheral blood from the patient, her parents and healthy controls. Reactions were set up using one set of primers upstream and downstream of exon 1 in triplicate. The β-actin gene served as a reference gene. The 2^−ΔΔ*CT*^ method was used to calculate the copy number. Samples with normalized ratios (NRs) <0.1 denote individuals with homozygous deletion, samples with NRs of about 0.5 denote individuals with heterozygous deletion, sample with NRs of ~1 denote healthy individuals (two copies), and samples with NRs of ~1.5 or more denote individuals with copy number gain.

To identify the genetic cause of the suspected primary immunodeficiency disease, whole-exome sequencing (WES) was performed using genomic DNA extracted from peripheral blood of the patient and her parents. A novel compound heterozygous mutation c.395T>G (p.Leu132Arg)/ex.1del (p.?) in the *IL-10RA* gene of the patient was identified, which was inherited from her mother and father. The p.Leu132Arg mutation is located on the “C” beta-strand within the D2 region of the extracellular domain of IL-10RA (10). *In silico* prediction tools predicted that the c.395T>G (p.Leu132Arg) missense variant was deleterious (SIFT score, −4.712; PolyPhen-2 score, 1.000), and it was defined as a likely pathogenic variant according to American College of Medical Genetics and Genomics (ACMG) guidelines (11). The ex.1del variant leads to a deletion of exon 1 of *IL-10RA* and is predicted to cause a loss of function of IL-10RA (SIFT score, −22.749). The ex.1del mutation was classified as a damaging variant according to ACMG guidelines. Furthermore, two *IL-10RA* variants were confirmed by Sanger sequencing (Figure 1A) or quantitative PCR (Figure 1B). Neither variant has been reported previously.

### Novel *IL-10RA* Mutation Causes an IL-10 Signaling Defect

**Figure 2:**
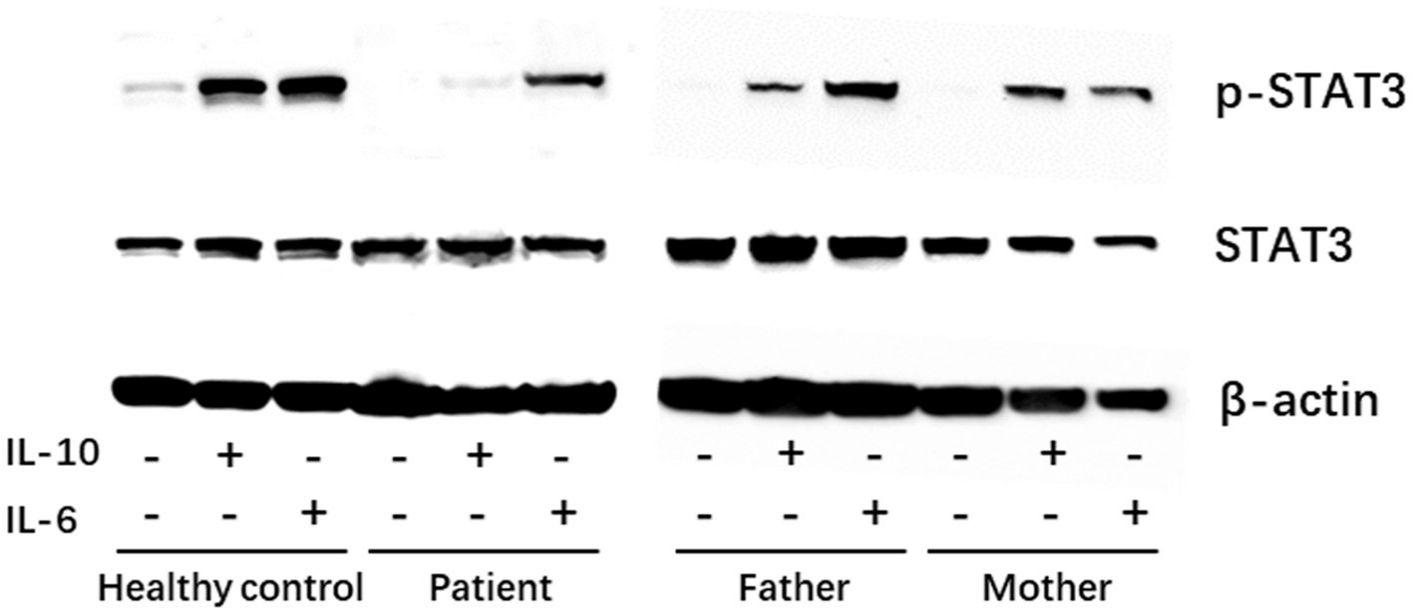
Western blot showing the patient had normal expression of p-STAT3 in peripheral blood mononuclear cells (PBMCs) in response to IL-6 (positive control) stimulation but showed defective STAT3 phosphorylation under IL-10 stimulation. −, untreated; +, treated.

To demonstrate a functional defect in the IL-10 signaling pathway caused by the identified novel *IL-10RA* mutation in the patient, PBMCs were isolated *via* Ficoll-Paque centrifugation and stimulated with recombinant human IL-6 (100 ng/ml) or IL-10 (100 ng/ml) for 30 min. Cell lysates were collected for immunoblotting to detect STAT3 phosphorylation. As shown in Figure 2, IL-10 treatment failed to activate STAT3 phosphorylation, whereas IL-6 induced the phosphorylation of STAT3 in PBMCs from the patient. Both IL-10 and IL-6 were able to induce the phosphorylation of STAT3 in PBMCs from healthy control and the patient's father and mother (Figure 2). Taken together, the patient was diagnosed with VEO-IBD caused by a novel compound heterozygous *IL-10RA* mutation.

### Hematopoietic Stem Cell Transplantation

After a diagnosis of VEO-IBD caused by IL-10RA deficiency, the patient was recommended for HSCT. During a long period of 10 months of management before HSCT, the patient presented with persistent symptoms of fever, abdominal pain, diarrhea, skin rashes, vulvar abscesses, and oral and perianal ulcers. The patient was admitted to our department repeatedly and was managed with symptomatic treatments, including broad-spectrum antibiotics for bacterial infection, acyclovir for viral infection, methylprednisolone for intestinal inflammation, thalidomide for immunosuppression, montmorillonite powder for diarrhea and nutritional support with TPN. At the age of 19 months, the patient underwent matched unrelated peripheral blood HSCT using reduced-intensity conditioning with busulfan, fludarabine, cyclophosphamide, and antithymocyte globulin. The CD34 cell dose was 7.5 × 10^6^ cells/kg, the number of mononuclear cells was 15.39 × 10^8^ cells/kg, and the platelet count was 202 × 10^9^/L. The patient was given methotrexate (3.5 mg/day) and cyclosporine A (40 mg/day) for 4 days to prevent graft-vs.-host disease. On day +14 post-HSCT, the short tandem repeat test of the blood showed that she engrafted with 94.6% of the donor cells. Currently, the patient's clinical manifestations are dramatically improved, including relieved abdominal pain and diarrhea (<4 times per day) and full recovery of skin papules, vulvar abscesses and perianal ulcers after 1 month of HSCT.

## Discussion

Although the exact etiology of IBD remains unknown, increasing evidence has shown that genetic predisposition plays an important role in the pathogenesis of IBD. In recent decades, over 250 IBD susceptibility loci have been identified by genome-wide association studies (GWASs); however, evidence of the causality of the majority of these loci is absent (3). Compared with the polygenic forms of IBD in older populations, the aggressiveness of the disease and young age of onset present a more significant genetic contribution to the development of VEO-IBD (12). Currently, more than 50 monogenic defects have been identified in patients with VEO-IBD, including gene mutations impairing intestinal epithelial barrier function, bacterial recognition and clearance, hyperimmune or autoimmune inflammatory pathways, and adaptive immune system function (3, 12). In this report, we described the case of a female VEO-IBD patient with initial disease onset at the age of 3 months. The patient suffered from recurrent episodes of unexplained fever, abdominal pain, diarrhea, and failure to thrive. WES identified a novel compound heterozygous mutation, c.395T>G (p.Leu132Arg)/ex.1del (p.?), in the *IL-10RA* gene of the patient. In addition, the pathogenicity of this novel mutation was confirmed by an IL-10RA function test through STAT3 phosphorylation analysis.

As a potent anti-inflammatory cytokine, IL-10 plays an important role in maintaining the balance of the immune system by suppressing the secretion of proinflammatory cytokines (13). IL-10 exerts its effect by binding to IL-10R, which is a tetrameric complex composed of 2 molecules of IL-10RA and 2 molecules of IL-10RB that activates the JAK1/STAT3 cascade and subsequently limits the expression of proinflammatory genes (13). Glocker et al. (14) identified three distinct homozygous mutations in the *IL-10RA* and *IL-10RB* genes of four children with VEO-IBD, and these were the first genes to be identified as causative for VEO-IBD. To date, the cases of ~150 IBD patients of various ethnicities with IL-10 signaling pathway defects have been described in the literature (15–17). Using a candidate gene sequencing approach, Kotlarz et al. (18) reported 16 VEO-IBD patients of different ethnicities (five Arabian, three Caucasian, two Kurdish, two Turkish, one Kurdish-Turkish, one Black, one Latin American, and one South Asian) with IL-10 or IL-10R deficiency (three patients had mutations in *IL-10*, five had mutations in *IL-10RA*, and eight had mutations in *IL-10RB*). Shim et al. (19, 20) reported seven cases of Korean infantile-onset IBD with *IL-10RA* mutations. Furthermore, Rahmani et al. (17) described 4 patients in a cohort of 25 Iranian patients with infantile-onset IBD who had missense mutations in *IL-10RA*, and one had a large deletion in *IL-10RB*.

Based on a large cohort study in China, Zheng et al. (15) analyzed the phenotypes of 139 reported VEO-IBD cases with identified *IL-10, IL-10-RA*, or *IL-10RB* mutations worldwide. It was shown that the majority of patients with IL-10 signaling defects had an extremely early disease onset age of <6 months and typically presented with perianal lesions (15). The most common extraintestinal manifestations were oral ulcers and skin rash (15). In agreement with those findings, the initial disease onset age of our reported patient was 3 months, and perianal lesions, oral ulcers, and skin rash were observed. Although *IL-10, IL-10RA*, and *IL-10RB* mutations were identified, *IL-10RA* mutations were more common than *IL-10* and *IL-10RB* mutations, particularly in patients from East Asia, including China, Japan, and South Korea. In Asian patients with IL-10RA mutations, c.C301T (p.R101W) and c.G537A (p.T179T) were the most common variants, and they were significantly more prevalent in Asian patients than in patients from other regions (15). We identified a novel compound heterozygous mutation, c.395T>G (p.Leu132Arg)/ex.1del (p.?), in *IL-10RA* that caused IL-10RA signaling defects in a Chinese child with VEO-IBD, which further expands the mutational spectrum of *IL-10RA*.

Currently, there is no standardized therapeutic approach to VEO-IBD. Symptomatic therapies are mostly used to treat VEO-IBD, including antibiotics, immunosuppressive drugs, anti-tumor necrosis factor-α agents and surgery (15). To date, HSCT is the sole curative therapy option for VEO-IBD that can achieve full recovery when performed at an early stage of the disease (15). The cases of 43 VEO-IBD patients with IL-10 signaling defect (34 with *IL-10RA* mutation, 6 with *IL-10RB* mutation, 2 with *IL-10* mutation, and 1 with *IL-R* mutation) underwent HSCT were reported (15, 21). The age at HSCT ranged from 4 months to over 16 years (15). The age at HSCT ranged from 4 months to over 16 years (15). The patients could achieve sustained clinical remission after HSCT, however, a high mortality rate (18.6%, 8/43) was observed (15, 21). Septic shock, graft failure, and idiopathic pneumonia syndrome are severe complications from HSCT that can cause death (15). Thus, future studies are needed to refine transplantation procedures to reduce the risks of HSCT. In this report, the patient's clinical manifestations were dramatically improved 1 month after HSCT, but the long-term effect of HSCT on the patient needs further evaluation.

In summary, we report the case of a Chinese child with VEO-IBD who carries a novel compound heterozygous mutation in *IL-10RA*, and the pathogenicity of the variant was confirmed by an IL-10RA function test through STAT3 phosphorylation analysis.

## Ethics Statement

This study was in compliance with the Helsinki Declaration and was approved by the Ethical Review Board of Shanghai Children's Hospital. Written informed consent to participate in this study was provided by the participants' legal guardian/next of kin. Written informed consents were obtained from the parents of the patients for the publication of this study. The parents of the patients consented to the publication of the case and any accompanying images with written consent.

## Author Contributions

TZ and YW conceived the study and edited the manuscript. FD and YW drafted the article. FD, FX, TG, XL, WX, and SW acquired, analyzed, and interpreted the data. All authors agreed to be accountable for all aspects of the work.

## Conflict of Interest

The authors declare that the research was conducted in the absence of any commercial or financial relationships that could be construed as a potential conflict of interest.
